# Effect of auricular point pressing therapy on hyperplasia of mammary glands: A protocol for systematic review and meta-analysis

**DOI:** 10.1097/MD.0000000000024875

**Published:** 2021-04-02

**Authors:** Mengjie Ma, Liuqiao Zhang, Xiangli Wang

**Affiliations:** Henan University of Traditional Chinese Medicine, 156 Jinshui East Road, Zhengdong New District, Zhengzhou City, Henan Province, China.

**Keywords:** auricular point pressing, hyperplasia of mammary glands, meta-analysis, systematic review

## Abstract

**Background:**

In recent years, with the accelerated pace of life, diet, environmental problems occur frequently. External factors are easily to cause endocrine disorders and hormone sensitivity of breast tissue, which can lead to mammary hyperplasia. The incidence rate of hyperplasia of mammary glands is increasing year by year, and the age of onset is also getting lower and lower. If not treated in time, there is a crisis of breast cancer.

Clinical studies have found that auricular point pressing therapy is widely used in clinical treatment of mammary hyperplasia recently, but the efficacy of massage in the treatment of mammary hyperplasia has not been systematically reviewed. The purpose of this study is to explore the efficacy, safety and effectiveness of auricular point pressing therapy in the treatment of hyperplasia of mammary glands.

**Methods:**

We will search PubMed, Web of Science, Cochrane Library, EMBASE, Wan fang Database, Chinese Scientific Journal Database, CNKI, VIP, and Chinese Biomedical Literature Database. The retrieval date was January 10, 2021. RevMan 5.3 software was used to evaluate the quality and risk of included studies. The efficacy, recurrence rate, and symptom score of breast hyperplasia were analyzed, and the results were observed and measured.

**Results:**

This study will be from the clinical efficiency, improvement rate, pain symptoms disappear rate, tumor size improvement rate, and other aspects of the existing evidence for a high quality synthesis, as well as auricular point pressing therapy adverse events.

**Conclusion:**

the conclusion of this review will provide the basis for judging whether auricular point pressing therapy is safe and effective in the treatment of hyperplasia of mammary glands.

**Ethics and dissemination:**

This systematic will evaluate the effectiveness and safety of auricular point pressing therapy in the treatment of hyperplasia of mammary glands. As all data used in this systematic review and meta-analysis have been published, ethical approval is not required for this review.

**Protocol registration number:**

INPLASY202110028.

## 1 Introduction

Hyperplasia of mammary glands is a degenerative disease and progressive connective tissue growth caused by hyperplasia of mammary fiber and epithelial tissue.^[1]^ Mammary gland hyperplasia belongs to the category of “breast fetish” in traditional Chinese medicine, which is mainly caused by stagnation of liver qi, spleen injury caused by thought or imbalance of Chong and Ren, and stagnation of qi and blood stasis.^[2,3]^ Studies show that breast hyperplasia is the highest incidence rate of female breast diseases, and has a certain correlation with the menarche time, the number of fetal birth, social economic status, and education level. About 75% of women have a certain degree of breast hyperplasia, and about 20% of women will be troubled by their clinical symptoms, among which 25 to 45 years old women have the highest incidence rate.^[4]^ Atypical hyperplasia is a precancerous lesion. The incidence rate of breast hyperplasia is also increasing with the increase of the disease course. According to the literature statistics, the canceration rate is between 1.25% and 50% then the HMG is a global health problem for women.^[5]^

Auricular point therapy of Traditional Chinese Medicine(TCM) is one of the external therapies with the characteristics of TCM, including pressing, acupuncture, bloodletting, needle imbedding, and other operations.^[6–8]^ According to TCM, auricular points are specific acupoints distributed on the auricle of human body, and they are the reaction points of pathological changes of Zang-fu Organs and meridians and collaterals on body surface.^[9,10]^ According to bioelectricity theory, the abnormal bioelectricity in pathological changes of the human body can cause the decrease of the resistance of corresponding auricular points. Related studies proved that after, 1 to 2 weeks of applying voltage to the auricle skin of peritonitis rats, the low resistance state of the skin around auricle improved.^[11]^ Clinically, auricular point pressing is the most commonly applied auricular point stimulation method at present, with advantages of long-lasting effect, simple operation, and less side effects.^[12]^ Clinical reports proved that this therapy plays a positive role in the intervention of headache, insomnia, and neurasthenia.^[13–15]^ At present, the effect of auricular point pressing therapy on hyperplasia of mammary glands lakes metaanalysis, thus hindering the spread of evidence. Therefore, according to the objective clinical needs, this study conducted a systematic review and meta-analysis of randomized controlled trials (RCTs) on the effect of auricular point pressing therapy on hyperplasia of mammary glands, so as to provide support for the spread of evidence.

## 2 Methods

The protocol has been registered on the INPLASY website, and the registration number is INPLASY202110028 (DOI number is 10.37766/inplasy2021.1.0028). The ethical approval and patient informed consent are abandoned because this study is based on published or registered RCTs.

### 2.1 Eligibility criteria

#### 2.1.1 Study designs to be included

We will only include randomized controlled trial (RCTs), non-RCTs, quasi-RCTs, reviews, and other types of studies will be excluded.

#### 2.1.2 Participant or population

Regardless of nationality, age, gender, occupation, participants met the clinical diagnostic criteria for breast hyperplasia were included. Cases associated with serious illness, pregnancy, lactation period were excluded.

#### 2.1.3 Intervention

The experimental group was treated with auricular point pressing therapy. The control group was treated with placebo, drugs, or other alternative therapy.

#### 2.1.4 Comparator

There is no exclusion based on comparator method for this review, and the patients could be treated with any forms of control group.

#### 2.1.5 Type of outcome

##### 2.1.5.1 Main outcome(s)

The main criteria are:

1. complete disappearance of pain symptoms;
2. diameter and area of breast mass;
3. hormone levels of luteinizing hormone, 17aestradiol, prolactin, and progesterone

##### 2.1.5.2 Additional outcome(s)

1. abnormal menstruation;
2. mood changes;
3. adverse reaction;
4. patient satisfaction.

### 2.2 Information sources

PubMed, Web of Science, Cochrane Library, EMBASE, Wan fang Database, Chinese Scientific Journal Database, CNKI, VIP, and Chinese Biomedical Literature Database were systematically searched by computer. The retrieval date was up to January 10, 2021. The details of PubMed's search strategy are illustrated in Table 1, while similar search strategies are applied for other electronic databases.

**Table 1:** Search strategy used in PubMed database.

Number	Search terms
#1	Hyperplasia of mammary glands[Title/Abstract]
#2	Breast hyperplasia[Title/Abstract]
#3	or/1-2
#4	Auricular[Title/Abstract]
#5	Point[Title/Abstract]
#6	Points[Title/Abstract]
#7	or/5-6
#8	#3 and #4 and #7
#9	Randomized controlled trial[Title/Abstract]
#10	Random trials[Title/Abstract]
#11	Controlled clinical trial[Title/Abstract]
#12	#9 or #11 #12

### 2.3 Study selection and management

**Figure 1:**
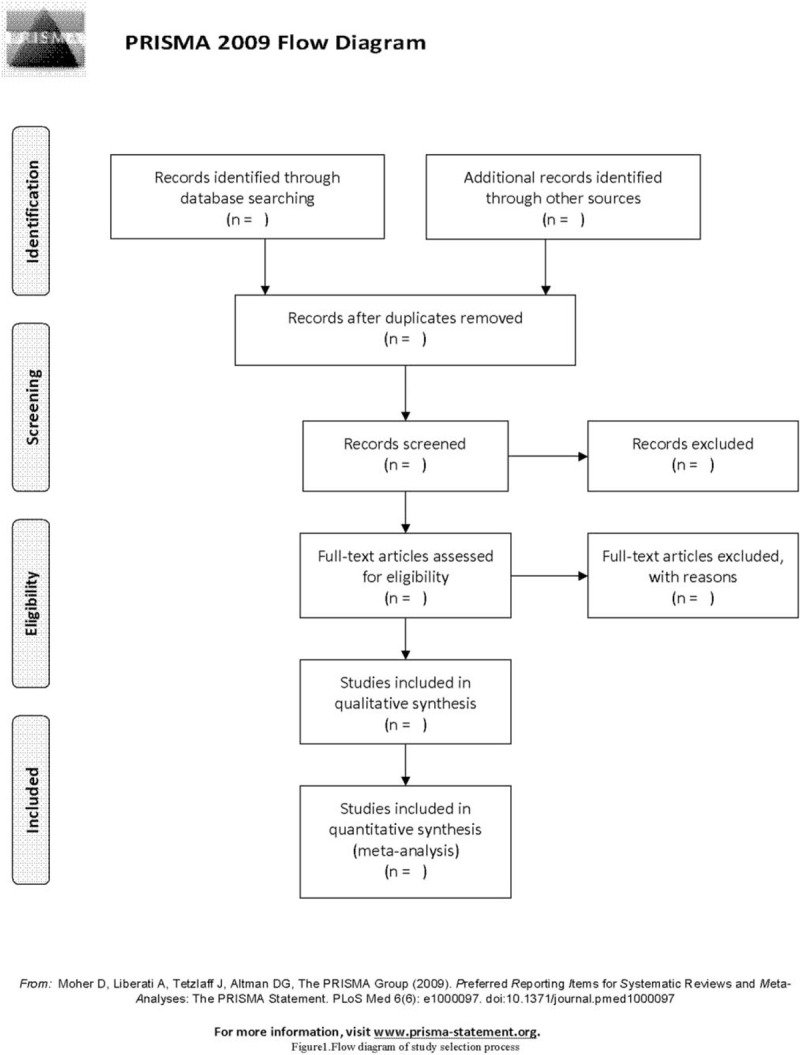
Flow diagram of study selection process.

According to the inclusion and exclusion criteria, firstly, the 2 researchers (M-MJ and W-XL) independently selected the literature after reading the titles and abstracts. Second, by reading the full text, we exclude uncontrolled studies, inconsistent evaluation criteria, and similar data. If there is any difference during the screening study, the third author (Z-LQ) will be involved. The screening flow chart of this study is demonstrated in Figure 1.

### 2.4 Data extraction and management

Two researchers (M-MJ and Z-LQ) will be charged of the data extraction according to the Cochrane Handbook for Systematic Reviews of Intervention. Including the title, first author, year, sample size, treatment process, intervention measures, outcome indicators, adverse reactions, etc.

### 2.5 Dealing with missing data

If the data is missing, we will contact the author to request the original data. If we cannot get the lost data, we will exclude them from the analysis.

### 2.6 Quality assessment/risk of bias analysis

The quality evaluation of literatures is based on Cochrane handbook system evaluation manual. The items of quality including random sequence generation method, allocation concealment, blinding of subjects, and intervention providers, blinding of outcome evaluators, completeness of result data, selective result reporting, and other sources of bias. According to the criteria, the included studies were divided into 3 levels: low, medium, and high.

### 2.7 Strategy of data synthesis

The Revman 5.3 software will be used to perform all statistical analyses and the heterogeneity was detected by Q test and *I*^*2*^ test. If *P* > .1, *I*^*2*^ < 50%, there is no significant heterogeneity between the included studies, fixed effect model will be used; If *P* < .1 and *I*^*2*^ ≥ 50%, it indicates that there is obvious heterogeneity among the included studies. We will use random effect model and subgroup analysis, sensitivity analysis to analyze the sources of heterogeneity to eliminate its impact.

### 2.8 Assessment of reporting biases

If more than 10 studies are included, a funnel chart would be utilized to assess the report bias.^[16]^ In addition, publication bias was further quantitatively evaluated by Egger and Begger test.

### 2.9 Assessment of heterogeneity

#### 2.9.1 Sensitivity analysis

For the quality analysis, we will conduct a sensitivity analysis of main outcomes to test the stability of the results of meta-analysis.

#### 2.9.2 Subgroup and metaregression analysis

When there is obvious heterogeneity (such as age, gender, different types of intervention, publication year, etc.), subgroup analysis and metaregression analysis will be conducted to identify the sources of heterogeneity.

## 3 Discussion

Oral drugs, especially hormone drugs, have certain side effects in the treatment of mammary hyperplasia at present. Western medicine or Chinese patent medicine often stimulate the gastrointestinal tract, and long-term medication will produce adverse reactions. Patients often relapse due to daily factors such as high pressure, poor sleep, mood fluctuation, and so on, and the recurrence rate is high. Although the acupuncture and catgut embedding therapy are effective, there are still some limitations in patients’ fear of acupuncture and syncope.^[17]^ Therefore, to explore more effective and safe treatment and improve the prognosis of patients with mammary hyperplasia has become an important health problem. Auricular plaster therapy in TCM is a traditional and common therapy for Non drug therapy.^[18]^ It is easy to operate, without injury and rare adverse reactions.^[19,20]^ It can be used to relax the tendons and disperse the knot, so that the meridians can be infused normally and promote the blood circulation. It has the advantages of rapid onset, simple, and convenient, short course of treatment, and easy to be accepted by patients.

The purpose of this study was to systematically review and evaluate all RCTs of the effect of auricular point pressing therapy on hyperplasia of mammary glands. The systematic review and meta-analysis of this article provide convincing conclusions for the efficacy and safety of auricular point pressing therapy for the treatment of hyperplasia of mammary glands. In addition, this study helps clinical doctors and nurses to treating breast hyperplasia, benefit patients, and provide reliable reference for its wide application.

## Author contributions

**Conceptualization:** Mengjie Ma.

**Data curation:** Liuqiao Zhang.

**Formal analysis:** Mengjie Ma, Xiangli Wang.

**Investigation:** Liuqiao Zhang.

**Methodology:** Mengjie Ma.

**Project administration:** Liuqiao Zhang.

**Software:** Mengjie Ma.

**Writing – original draft:** Mengjie Ma, Liuqiao Zhang.

**Writing – review & editing:** Mengjie Ma, Xiangli Wang.
